# Current Knowledge and Future Research Directions on Fecal Bacterial Patterns and Their Association with Asthma

**DOI:** 10.3389/fmicb.2016.00838

**Published:** 2016-06-29

**Authors:** Shantelle Claassen-Weitz, Charles S. Wiysonge, Shingai Machingaidze, Lehana Thabane, William G. C. Horsnell, Heather J. Zar, Mark P. Nicol, Mamadou Kaba

**Affiliations:** ^1^Division of Medical Microbiology, Department of Pathology, Faculty of Health Sciences, University of Cape TownCape Town, South Africa; ^2^Centre for Evidence-based Health Care, Faculty of Medicine and Health Sciences, Stellenbosch UniversityCape Town, South Africa; ^3^Cochrane South Africa, South African Medical Research CouncilCape Town, South Africa; ^4^Vaccines for Africa Initiative, Institute of Infectious Disease and Molecular Medicine, University of Cape TownCape Town, South Africa; ^5^Department of Clinical Epidemiology and Biostatistics, McMaster UniversityOntario, Canada; ^6^Biostatistics Unit, Father Sean O'SulliVan Research CentreOntario, Canada; ^7^Division of Immunology, Department of Pathology, Faculty of Health Sciences, University of Cape TownCape Town, South Africa; ^8^Institute of Infectious Diseases and Molecular Medicine, University of Cape TownCape Town, South Africa; ^9^International Centre for Genetic Engineering and Biotechnology, University of Cape TownCape Town, South Africa; ^10^Department of Paediatrics and Child Health, University of Cape TownCape Town, South Africa; ^11^Red Cross War Memorial Children's HospitalCape Town, South Africa; ^12^Medical Research Council Unit on Child and Adolescent Health, University of Cape TownCape Town, South Africa; ^13^National Health Laboratory Service, Groote Schuur HospitalCape Town, South Africa

**Keywords:** asthma, fecal bacteria, mechanisms, microbiome, systematic review

## Introduction

Asthma is a complex respiratory condition that involves interplay between genetic predisposition, environmental, and immunological factors (Edwards et al., 2012). It is considered to be one of the most common chronic diseases, affecting ~300 million people (Masoli et al., 2004), and causing an estimated 250,000 deaths annually (Bateman et al., 2008). Furthermore, because of an increased Westernized lifestyle and urbanization in developing countries, it is estimated that by 2025 the global burden of asthma will increase by 100 million people (Masoli et al., 2004).

An increase in the occurrence of allergic diseases, including asthma, was initially attributed to the “hygiene hypothesis,” suggesting that a reduced exposure to microbes during the first years of life plays a role in the development of allergic diseases (Strachan, 1989, 2000). Although this hypothesis is widely accepted, studies showed that reduced microbial exposure cannot fully account for the increased prevalence of asthma, rhinitis, or neurodermitis (Mallol, 2008; Brooks et al., 2013; Kramer et al., 2013). Alternative hypotheses or reformulations of the “hygiene hypothesis” (Hunter, 2012), such as the “microbiota hypothesis” (Wold, 1998), the “old friends hypothesis” (Rook, 2012), the “microbial deprivation hypothesis” (Bloomfield et al., 2006), the “biodiversity hypothesis” (Hanski et al., 2012) and the “disappearing microbiota hypothesis” (Blaser and Falkow, 2009; Taube and Müller, 2012) soon followed; all of which mainly postulate that dysbiosis of the human gastro-intestinal tract (GIT) microbiome may contribute to intra- and extra-intestinal immune-mediated diseases (Penders et al., 2007; Štšepetova et al., 2007; Sekirov et al., 2010; Clemente et al., 2012; Russell and Finlay, 2012). Understanding which bacteria from our GITs contribute to the development or prevention of allergic asthma may result in further research to discern the mechanisms behind bacterial-host interactions and potentially facilitate treatment strategies.

## Methods used to study the role of human fecal bacteria in asthma

Although culture-independent techniques have revolutionized the world of microbiology (Suau et al., 1999; Zoetendal et al., 2006; Rajilić-Stojanović et al., 2007); conventional culture-dependent techniques have been the method widely used to study the role of human fecal bacteria in asthma (Mansson and Colldahl, 1965; Stockert, 2001; Nambu et al., 2008; Vael et al., 2008; Bisgaard et al., 2011). To date, the culture-independent techniques used to characterize fecal bacteria from patients with asthma include quantitative real-time polymerase chain reaction (qPCR) (Van Nimwegen et al., 2011), denaturing gradient gel electrophoresis (DGGE) (Bisgaard et al., 2011; Vael et al., 2011), fluorescent *in situ* hybridization (FISH) (Salminen et al., 2004), and massively parallel high-throughput sequencing of the 16S ribosomal RNA (rRNA) gene (Arrieta et al., 2015). Despite the advantage of detecting uncultivable bacteria, these culture-independent techniques are not without limitations. Among others, they do not allow for whole community analysis of the microbial population (Sekirov et al., 2010; Fraher et al., 2012; Sankar et al., 2015), which is considered key in determining the patterns of fecal bacteria associated with health and disease states (Schippa and Conte, 2014). For example, qPCR and FISH do not provide identification of novel organisms as they are used to characterize and quantify targeted groups of bacteria (Sekirov et al., 2010; Fraher et al., 2012). DGGE, a band-based method for determining bacterial diversity, does not enable direct identification of bacteria (Sekirov et al., 2010; Fraher et al., 2012). Furthermore, DGGE has low bacterial detection limits and limited phylogenetic resolution (Sekirov et al., 2010). Although massively parallel high-throughput sequencing of the 16S rRNA gene provides an almost comprehensive view of bacterial communities; it does not provide classification at species-level (Gosalbes et al., 2012; Arrieta et al., 2015). The importance of species-level characterization in health and disease states has been demonstrated in murine models of allergic diseases (Karimi et al., 2009; Russell et al., 2012; Kim et al., 2013). An overgrowth of the genus *Lactobacillus* has been associated with an increased risk of allergic asthma (Russell et al., 2012), while the species *L. reuteri* and *L. rhamnosus* provide a protective role in allergic airway disease (Karimi et al., 2009; Kim et al., 2013). In comparison to 16S rRNA gene sequencing techniques; whole genome shotgun (WGS) sequencing offers a higher and more reliable resolution of microbiota profiles at lower taxonomic levels (Morgan and Huttenhower, 2014; Van Dijk et al., 2014; Ranjan et al., 2015). For example, WGS sequencing is able to improve the issue related to the *Bifidobacterium* amplification bias by certain primer sets (Kurokawa et al., 2007; Sim et al., 2012; Walker et al., 2015). In addition, it allows for determination of the metabolic and functional properties of fecal bacteria which may greatly contribute to our understanding of the role of fecal bacteria in health and disease (Qin et al., 2012; Arrieta et al., 2015; Quince et al., 2015). However, despite the number of advantages that WGS sequencing provides, it has not been incorporated by any of the studies investigating the importance of fecal bacteria in the development of asthma. Furthermore, a causal link between fecal bacterial profiles and asthma in humans has recently been confirmed using murine models (Arrieta et al., 2015). To the best of our knowledge, this is the only report of its kind where the causal role of the fecal bacteria (*Lachnospira, Veillonella, Faecalibacterium*, and *Rothia*), which potentially confers protection against the development of asthma in humans, was demonstrated using germ-free mice models (Arrieta et al., 2015).

## What do studies in humans reveal about the role of fecal bacteria in asthma?

Although prospective longitudinal studies are key in demonstrating the role of fecal bacteria in disease development (Zhao, 2013); we identified only two studies which assessed whether fecal bacterial profiles sampled over time preceded the occurrence of asthma at later stages in life (Bisgaard et al., 2011; Arrieta et al., 2015). Bisgaard et al. (2011) did not report a significant association (Bisgaard et al., 2011). In contrast, Arrieta et al. (2015) found significantly reduced abundances of the bacterial genera *Lachnospira, Veillonella, Rothia*, and *Faecalibacterium* in infants at risk for asthma, as evidenced using the Asthma Predictive Index (API) (Arrieta et al., 2015). Moreover, in a prospective birth cohort study conducted in Belgium (using fecal specimens sampled at 3 weeks of age); the detection of *Bacteroides* (*B. fragilis, B. finegoldii*, and *B. thetaiotaomicron*)*, Ruminococcus* (*R. productus* and *R. hansenii*), and *Clostridium* spp. was associated with an increased risk for asthma development (as based on the API) (Vael et al., 2011). At species-level, the prospective birth cohort study by Van Nimwegen et al. (2011) conducted in the Netherlands reported a two-fold increased risk of asthma at 6–7 years in infants colonized with *Clostridium difficile* at 1 month of life (OR = 2.06; 95% CI 1.16–3.64) (Van Nimwegen et al., 2011).

All prospective birth cohort studies, except for the study by Nambu et al. (2008), made use of the API when assessing asthma as an outcome at < 5 years of age (Vael et al., 2008, 2011; Arrieta et al., 2015). The API, incorporated by three studies cited in this review, is an example of a predictive assessment for asthma development later in life, recommended for young children experiencing recurrent wheeze (Castro-Rodriguez, 2010). Considering that asthma diagnosis in children < 5 years of age is challenging and often based on symptom patterns, clinical assessment of the family history and the presence of atopy (Pedersen, 2007; Sly et al., 2008; Pedersen et al., 2011); the use of predictive assessments, such as the API, is essential. However, despite its success in developed countries, the API should be used with caution in infants from low and middle income countries (LMICs) (Zar and Levin, 2012). This may be explained by the fact that young children from LMICs are more commonly affected by viral lower respiratory tract infections (LRTIs) or pulmonary tuberculosis. Furthermore, it has been suggested that atopy may be less strongly associated with asthma in these settings compared to the more developed countries (Zar and Levin, 2012). This suggests that non-atopic wheeze may be the primary form of asthma in these children, making the API, which relies primarily on the presence of atopy for assessing the risk of asthma, a less reliable predictive assessment tool in LMICs (Zar and Levin, 2012).

## Factors influencing fecal bacterial profiles and potentially asthma

Both murine models and human studies have provided evidence that early life changes in the GIT microbiome are most influential in the development of allergic asthma (Russell et al., 2013; Arrieta et al., 2015). Some of the well described factors responsible for these early life changes in fecal bacterial profiles, which have also been associated with childhood asthma, are mode of delivery, feeding practices, and antibiotic use (Kozyrskyj et al., 2011).

### Mode of delivery

A number of childhood studies have reported that infants delivered via cesarean section are at an increased risk for the development of asthma (Thavagnanam et al., 2008). However, these studies do not account for confounding factors that may be associated independently with asthma, as well as changes in fecal bacterial profiles, which will allow for determining the true effect of external factors on fecal microbiota and the resultant health outcome. To date, only a single study using mediation analysis (Van Nimwegen et al., 2011) supported the role of mode and place of delivery (independent variable) in *C. difficile* colonization (mediator variable), together with its consequent impact on asthma development via modulation of *C. difficile* profiles (dependent variable).

### Feeding practices

Although, it has been reported that breastfeeding has the potential to protect against allergic airway disease (Dogaru et al., 2014), no studies have used mediation analysis (as performed by Van Nimwegen et al. (2011)) to determine whether bacteria from breast milk protect against asthma via the modulation of infant fecal bacteria.

### Antibiotic use

In humans, a modest increased risk of asthma development, associated with antibiotic use, has been reported (Marra et al., 2009; Risnes et al., 2010; Murk et al., 2011; Penders et al., 2011). To date, only fecal *C. difficile* colonization has been associated with the occurrence of asthma (Van Nimwegen et al., 2011), which might be explained (among other factors) by a loss of intestinal commensal microbes through the use of antibiotics (Azad and Kozyrskyj, 2012).

## Potential mechanisms supporting the role of gastrointestinal bacteria in asthma

**Figure 1:**
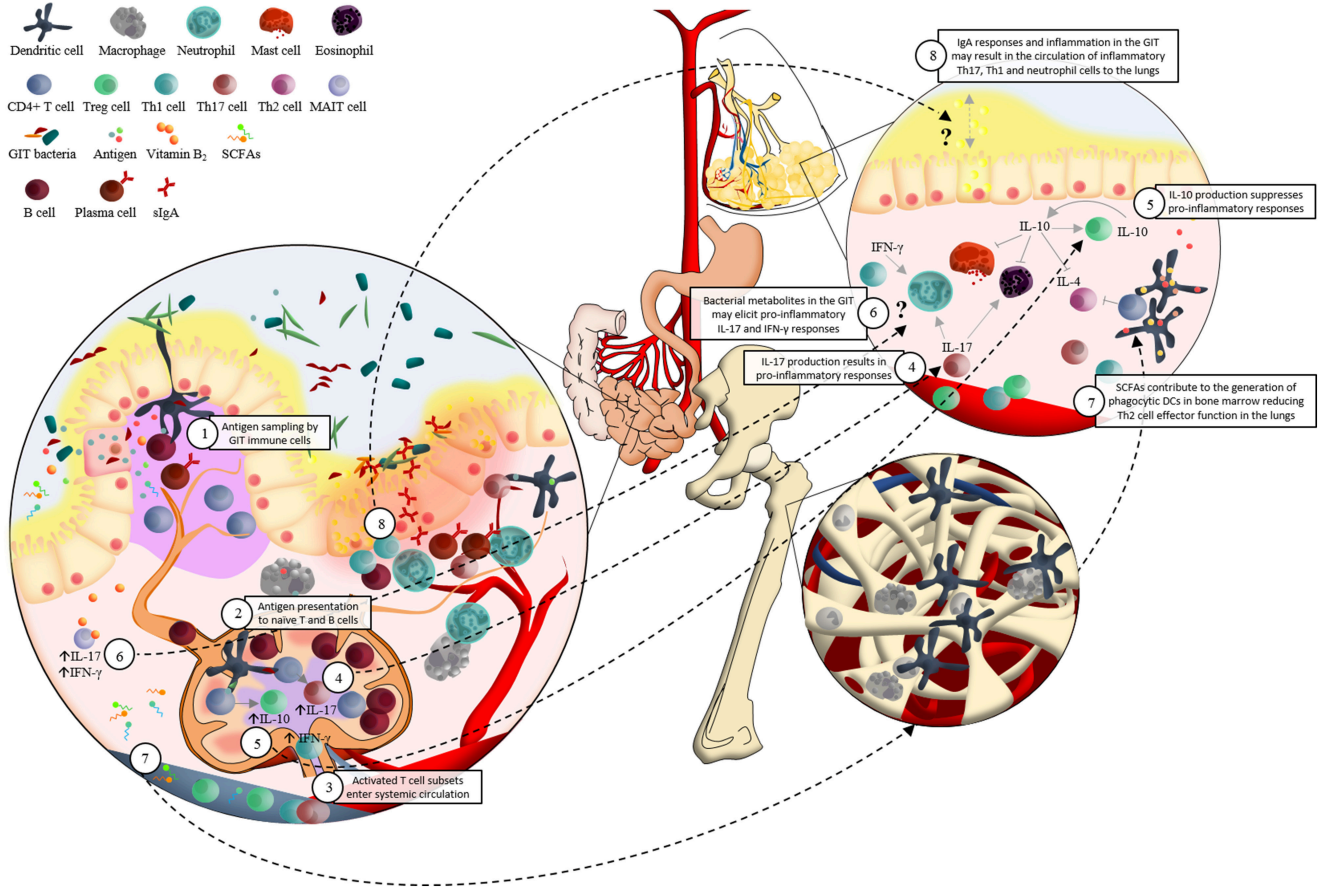
**Schematic representation of the potential immunological interaction between the gastrointestinal tract microbiota and the development of asthma**. 1. Dendritic cells (DCs) sample antigen in the lamina propria (LP) and Peyer's patches (PP) of the small intestine; and by extending their dendrites into the intestinal lumen. 2. The interactions between DCs and microbial associated molecular patterns (MAMPs) allow DCs to present antigen to naïve lymphocytes in the mesenteric lymph nodes (mLNs). For example, DCs present epitopes together with major histocompatibility complex (MHC) class II and specific immunomodulatory cytokines to naïve CD4+ T cells. This elicits proliferation and activation of various T cell subsets which 3. enter systemic circulation via the efferent lymph, homing to mucosal surfaces inside and outside of the gastrointestinal tract (GIT). 4. TGF-β contributes to the differentiation of Th17 cells, which produce cytokines (such as IL-17) involved in pro-inflammatory responses. 5. IL-12 associated cytokines are responsible for Th1 cell differentiation. This regulates the induction of IL-10, which supresses pro-inflammatory responses. 6. Presentation of vitamin B2, from a wide range of bacteria and fungi via MR1 molecules, to mucosa-associated invariant T (MAIT) cells results in a rapid production of pro-inflammatory Th1/Th17 cytokines. MAIT cells' preferential location in the GIT LP and PP, as well as their pro-inflammatory responses in reaction to bacterial metabolites such as vitamin B2, may support their potential role in asthma pathogenesis via the “gut-lung axis” in a similar manner to what has been proposed for DCs. 7. Circulating short-chain fatty acids (SCFAs) contribute to the protection against allergic airway inflammation via enhanced generation of DC precursors in bone marrow, followed by seeding of the lungs with DCs with high phagocytic capacity and limited ability to promote Th2 cell effector function. 8. Localization of inflammatory GIT bacteria in the GIT mucus layer may induce strong IgA responses and chronic local inflammation. An influx of inflammatory Th17, Th1, and neutrophil cells in the GIT could potentially circulate to the lungs where they may contribute to asthma pathogenesis. This hypothesis may be supported by the strong associations found between irritable bowel disease (IBD) and asthma.

The exact mechanisms by which GIT bacteria may influence the development of respiratory diseases are unclear; however recent work has demonstrated that crosstalk between host mucosal immune cells and resident microbes significantly influences the risk for respiratory disease (Forsythe, 2011; Samuelson et al., 2015; Vital et al., 2015). A central player in this regulation of pulmonary immunity by the GIT microbiome are dendritic cells (DCs) (McLoughlin and Mills, 2011) (Figure 1). Intestinal DCs encounter bacterial antigens presented in organized GIT immune tissue (i.e., lamina propria and Peyer's patches) and also directly sample lumen residing bacteria in the GIT by extending their dendrites into the intestinal lumen (Salzman, 2011). This sampling of intestinal bacterial antigens results in DCs co-ordinating B and T cell subset expansion both locally (Peyer's patches and lamina propria) as well as systemically (e.g mesenteric lymph nodes) (Hill and Artis, 2010) (Figure 1). This results in DC-guided local and systemic immune education driven by microbiota associated antigens which has profound effects not just in the intestine but throughout the body (Hill and Artis, 2010; Russell and Finlay, 2012) (Figure 1). An important consequence of this effect of GIT bacteria is manifested in subsequent host T-cell immune responses in the lungs and has been particularly well demonstrated in murine models of asthma (Herbst et al., 2011; Navarro et al., 2011; Konieczna et al., 2012; Oertli et al., 2012). For example, *B. fragilis* and *Clostridium* species (cluster IV and XIVa), both intestinally restricted bacteria, can drive induction of T regulatory (Treg) cells and associated elevated secretion of the regulatory cytokine IL-10 in mesenteric lymph nodes to mediate protection against allergic T-helper cell (Th-) 2 airway inflammation (Round et al., 2011) (Figure 1). Other studies have demonstrated that early life depletion of *Bacteroidetes* species using vancomycin abrogates the ability of mice to launch Treg protection from allergic asthma (Atarashi et al., 2011; Russell et al., 2012). In addition, raised levels of *Helicobacter pylori* has also been shown to elicit protection against the development of asthma, again, via the induction of Treg cells (Arnold et al., 2011). Interestingly this effect may, in part at least, also be due to *de novo* production of IL-10 orthologs by *H. pylori* driving this Treg induction. Moreover, oral administration of probiotics (*L. reuteri, L. rhamnosus GG, Bifidobacterium breve*, or *B. lactis*) can impair the onset of ovalbumin induced allergic airway inflammation; again related to the reduced induction of Treg cells (Feleszko et al., 2007; Karimi et al., 2009). Taken together, these and other studies are generating an important profile of the microbial species driving Treg dependent protection against allergic airway inflammation. Other studies have also identified bacteria which may drive the onset of allergic pathology. Segmented filamentous bacteria (SFB), non-cultivable Clostridia-related host-specific species (Gaboriau-Routhiau et al., 2009), and members of the cytophaga-flavobacter-bacteroides (CFB) phylum, for example, have been shown to promote differentiation of pro-inflammatory Th17 cells associated with airway inflammation (Ivanov et al., 2008; Atarashi et al., 2011) (Figure 1).

Although the studies described here provide insight into the potential role of GIT bacteria in the development of asthma in murine models; more studies are needed to explore the manner in which whole GIT bacterial communities, from asthmatic and non-asthmatic participants, interact with the innate and adaptive immune cells of the GIT, as well as their subsequent immune effects in the lungs. Besides, studies should also investigate a broader scope of mechanisms to explain the role of GIT bacteria in asthma pathogenesis. For example, a potential mechanism in need of further investigation is the tenable role of IgA-coated inflammatory GIT bacteria in the development of asthmatic responses in the lungs (Figure 1). To date, no clear link between host GIT microbiota-idiopathic intestinal inflammation and allergic lung disease has been demonstrated. However, our recent understanding of the involvement of IgA-coated bacteria in intestinal inflammation (Van der Waaij et al., 2004; Palm et al., 2014), as well as the number of clinical studies denoting an association between inflammatory lung disease and intestinal inflammation (Tulic et al., 2016); provides rationale for investigating the systemic immune effect of IgA-coated GIT bacteria. For example, it is suspected that around 50% of patients suffering from ulcerative colitis and Crohn's disease have subclinical pulmonary abnormalities with low-grade airway inflammation (Kuzela et al., 1999; Mohamed-Hussein et al., 2007). Moreover, a large cohort study, investigating 5260 IBD patients together with 21,040 non-IBD participants, recently provided strong evidence for the association between IBD and an increased risk for asthma (Peng et al., 2015). In support of this, Palm et al. (2014) clearly showed microbial localization of IgA positive bacteria from IBD patients in the normally sterile GIT mucosa of germ-free mice, which was not observed for IgA negative bacteria from IBD patients (Palm et al., 2014). We therefore hypothesize that GIT bacteria characterized by high levels of IgA coating may enter the GIT mucosa (Palm et al., 2014) where they may elicit systemic inflammatory responses at extra-intestinal mucosal sites such as the lungs.

In addition to assessing the immuno-regulatory effect of the composition of GIT bacteria in asthmatic and non-asthmatic participants; studies should also investigate the functional characteristics of GIT bacteria in the occurrence of asthma. In support of this, Trompette et al. (2014) reported the role of circulating short-chain fatty acids (SCFAs) in the protection against allergic airway inflammation (Trompette et al., 2014) (Figure 1). Here, microbiome metabolism in a high fiber diet setting resulted in enhanced SCFA metabolism leading to the generation of myeloid bone marrow precursors that gave rise to populations of pulmonary DCs that protected against Th2 driven allergic airway disease (Trompette et al., 2014). In support of this, Zaiss et al. (2015) demonstrated attenuated allergic airway inflammation via a GPR41 (SCFA receptor) dependent manner, as well as the effect of changes in GIT bacteria on SCFA production (Zaiss et al., 2015). Furthermore, microbial vitamin B_2_ (riboflavin) metabolites have been shown to activate a subset of innate-like T cells, the mucosa-associated invariant T (MAIT) cells, which are highly abundant in peripheral blood, mucosal tissues, as well as the liver (Treiner et al., 2003; Le Bourhis et al., 2013). Vitamin B_2_ from a wide range of bacteria and fungi are presented to MAIT cells by MR1 molecules (Kjer-Nielsen et al., 2012; Patel et al., 2013), followed by the rapid production of pro-inflammatory Th1/Th17 cytokines such as interferon-gamma (IFNγ) and IL-17 (Le Bourhis et al., 2011) (Figure 1). MAIT cells' pro-inflammatory responses in reaction to bacterial metabolites, together with their preferential location in the GIT lamina propria and mesenteric lymph nodes (Treiner et al., 2003), may support the “gut-lung axis” theory in a similar manner to what has been proposed for DCs. Therefore, functional properties of GIT bacteria such as dietary fiber metabolism and the production of vitamin B_2_ may be an important aspect of host microbe crosstalk.

## The potential of modulating gastrointestinal microbiota to protect against asthma

The mechanistic insights into how GIT bacteria may protect or contribute to the development of asthma have provided great potential for the development of intervention studies. For example, the administration of probiotics (beneficial live bacterial species) (De Kivit et al., 2014), prebiotics (non-digestible food ingredients) (Jeurink et al., 2013) or symbiotics (synergistic nutritional supplements combining probiotics and prebiotics) (Van de Pol et al., 2011; Van der Aa et al., 2011) have demonstrated immune-modulatory potential via the restoration of an altered intestinal microbiota. The efficacy of probiotic administration (mainly *Lactobacillus* or *Bifidobacterium* spp.) in treatment or prevention of asthma has clearly been demonstrated in animal models (Feleszko et al., 2007; Karimi et al., 2009; MacSharry et al., 2012; Kim et al., 2013); however data in humans are not conclusive (Vliagoftis et al., 2008; Elazab et al., 2013). Nevertheless, probiotic administration needs to be carefully considered as we do not fully understand its effect on GIT bacteria. It may also hold more complex effects for the host (Shenderov, 2013), such as infections (Fijan, 2014) and allergic sensitization (Viljanen et al., 2005; Taylor et al., 2007). Various factors therefore need to be taken into account in the development of probiotics. These include the immunological pathways behind immune responses elicited by live bacteria and bacterial molecules (Caselli et al., 2011); the ongoing research around what a “healthy” GIT profile should look like (Koren et al., 2013; Knights et al., 2014) (prior to considering modulation thereof); what the effect of probiotics are on these “healthy” GIT profiles (Eloe-fadrosh et al., 2015); the inter-individual variability of the human GIT microbiome (De Filippo et al., 2010; Grześkowiak et al., 2012; Yatsunenko et al., 2012; Lin et al., 2013; Ou et al., 2013; Suzuki and Worobey, 2014); the effect of probiotics on host metabolic and signaling pathways (Shenderov, 2013); and whether diversity within specific bacterial taxa is of importance in immunological tolerance (West, 2014). In addition, studies are needed to assess the period, dose and duration of probiotic supplementation. As for probiotics, prebiotic supplementation was not significantly associated with the prevention of asthma in humans (Arslanoglu et al., 2012; Osborn and Sinn, 2013); however, administration of oligosaccharides in mice has been associated with decreased parameters of allergic asthma (Vos et al., 2007).

It is important to also highlight the potential role of vitamin D in modulation of the GIT bacterial community and consequent immune responses such as asthma (Arshi et al., 2014). Vitamin D not only acts on a number of immune cells and processes involved in immune regulation of asthma (Brehm et al., 2009, 2010; Mann et al., 2014), but also has the potential to modulate GIT bacterial profiles and their functions (Mai et al., 2009; Jin et al., 2015). Thus, further exploring the therapeutic potential of vitamin D supplementation, together with pro-, pre- and synbiotic interventions, in modulating the host's GIT microbiota and its subsequent effect on allergic airway diseases such as asthma has merit.

Moreover, understanding the effects of other GIT microbiota, such as fungi and helminths, on the composition of GIT bacteria is also likely to be extremely informative. For example, an overgrowth of commensal fungal *Candida* species in the GIT, as a result of antibiotic treatment, has been shown to promote M2 macrophage activation in the lungs, as well as increased allergic airway inflammation (Kim et al., 2014). In addition, changes in the GIT bacterial composition of mice following chronic infection with the murine helminth *Heligmosomoides polygyrus bakeri* have been elegantly shown to protect against house dust mite induced airway inflammation (Zaiss et al., 2015). Importantly this study shows that these changes resulted in elevated SCFA production that actually underlies the protective phenotype (Zaiss et al., 2015). This and work discussed above from the Marsland laboratory provide strong evidence for dietary modulation of the microbiome protecting against allergy.

## Conclusion and perspectives

A systematic search of the literature revealed that studies investigating fecal bacteria from humans and their relationship with asthma have been increasingly published since the beginning of the 21st century. However, reports on the role of fecal bacteria in the development of asthma in humans are limited, and primarily investigate the role of select GIT bacteria in asthma pathogenesis. Large longitudinal prospective cohort studies, with clear definitions of asthmatic outcomes, incorporating high-resolution methods (such as massively parallel 16S rRNA gene sequencing, whole-genome shotgun sequencing or culturomics), are therefore needed to determine the role of fecal bacteria in the development of asthma in both developed and developing countries. Studies also need to assess the impact of covariates (such as mode of delivery and intestinal microbes other than bacteria) on both fecal bacterial profiles and the outcome of interest using rigorous statistical analyses. Furthermore, studies should aim to test the causal link between human fecal bacteria and asthma development using murine models. Finally, the role of GIT bacteria in asthma should be investigated alongside the airway microbiome in order not to mask the importance of the local respiratory microbial-host interactions. In addition, it would be interesting to assess whether GIT bacteria impacts on corticosteroid responsiveness in asthma (Goleva et al., 2013), as well as asthma severity and phenotypes (Zhang et al., 2016).

### Literature search strategy and selection criteria

We systematically searched peer-reviewed articles published on bacteria detected from feces and their association with asthma from six electronic databases (Medline via Pubmed, Scopus via SciVerse, Academic Search Premier, Africa-Wide Information and CINAHL via EBSCOHost and Web of Science via Web of Knowledge), using a combination of keywords [(microbiota^*^ OR metagenome OR microbiome^*^ OR “human microbiota^*^” OR “human microbiome^*^” OR “gut microbiota^*^” OR “gut microbiome^*^” OR “intestinal flora” OR “digestive flora” OR “gut flora” OR feces OR stool OR faeces OR fecal OR faecal) AND (asthma OR “bronchial asthma” OR “bronchial disease^*^” OR “respiratory sound^*^” OR “lung sound^*^” OR wheez^*^)]. The last literature search was 19 November 2015. All articles published in English and French were assessed for inclusion in the review. Original research articles investigating bacteria from fecal specimens and their relation to asthma in humans unexposed to antibiotic, pre- or probiotic treatments were included. In addition, we cross-checked the reference lists of all eligible studies included in this review for any additional articles.

## Author contributions

MK, SC, and MN initiated the project. SC extracted the data and reviewed the articles with MK. SC, CW, SM, LT, and MK performed the statistical analysis and interpreted the results. SC, LT, WH, HZ, MN, and MK wrote the manuscript.

## Funding

This work was supported by the Bill and Melinda Gates Foundation Global Health Grant (OPP1017641), the National Research Foundation (South Africa), the Carnegie Corporation of New York (United States of America), the US National Institutes of Health (1U01AI110466-01A1), and the Wellcome Trust, United Kingdom (102429/Z/13/Z). The first (SC) and the corresponding author (MK) had full access to the study data. All authors had final responsibility for the decision to submit the article for publication.

### Conflict of interest statement

The authors declare that the research was conducted in the absence of any commercial or financial relationships that could be construed as a potential conflict of interest.
